# Parental Awareness about Oral Health Preventive Care and its Relation to DMFT Index in Visually Impaired Children

**DOI:** 10.30476/DENTJODS.2019.80995.0

**Published:** 2020-06

**Authors:** Narjes Amrollahi, Andisheh Amini, Mehdi Jafarzadeh

**Affiliations:** 1 Dental Research Center, Dept. of Pediatric Dentistry, Dental Research Institute, Isfahan University of Medical sciences, Isfahan, Iran; 2 Dental Students' Research Committee, School of Dentistry, Isfahan University of Medical Sciences, Isfahan, Iran

**Keywords:** Dental caries, Knowledge, Visually impaired persons, Parents

## Abstract

**Statement of the Problem:**

Oral health care for children with visual impairment is challenging for health service providers. Gaining information about parental awareness in this regard can be a basis for health planning and use of preventive services.

**Purpose:**

The aim of this study was to evaluate parental awareness about the importance of preventive care and its relationship with DMFT index in visually impaired children.

**Materials and Method:**

This cross-sectional and descriptive-analytical study was carried out on 50 parents of children with visual impairment referring to schools of the blind children aged 8-14 years in Isfahan in 2017-2018. Parental awareness was measured by knowledge questionnaire. DMFT index of first permanent molar was recorded by examination. Data were analyzed using Spearman correlation coefficient, Pearson correlation and T-test in SPSS 22 software. The level of significance was considered less than 0.05.

**Results:**

The mean score of parental awareness about significance of preventive care was 68.4±15.5. Mean DMFT in the studied children was 2.40±1.32 and highest score was related to decayed tooth, followed by filled tooth. There was no significant relation between parental knowledge and mean DMFT (*p*= 0.30), while there was revers relation with number of extracted teeth (p= 0.02) and direct relation with number of filled tooth (*p*= 0.04).

**Conclusion:**

Parental awareness generally did not show significant relationship with DMFT, while by increasing their knowledge the number of missed teeth was decreased and the number of filled teeth was increased in visually impaired children.

## Introduction

Oral health is a substantial aspect of health for all children, especially in disabled children and responsibility of oral hygiene care is by parent or guardian, that many of them have poor information in this regard. Visually impaired children have some difficulties to achieve an ideal oral hygiene due to their disability [ 1 - 2 ]. These children are often unable to apply oral hygiene instructions and plaque control [ 3 ]. Also, blind children are more likely to develop dental caries and inflammatory disease of the periodontium. Because they are unable to visualize the plaque on the tooth surfaces and the early evidence of dental caries including color changes in the teeth [ 4 ].

In recent years, very little attention has been paid to the dental health of the visually impaired children, who deserve special care in this regard. Although extensive studies [ 1 , 4 - 7 ] have been done in physically disabled children, no data regarding the oral hygiene status for visually impaired children are available in Isfahan city.

Gaining information on parental awareness in this area can be the basis for health planning and the use of preventive services. Moreover, this information can assist health providers in implementing oral health promotion programs among the visually impaired children. In the current study, parental awareness about the importance of preventive care and its relation with DMFT is investigated.

## Materials and Method

This cross-sectional and descriptive- analytical study was approved by the Ethics Committee of Isfahan University of Medical Sciences, Isfahan, Iran (ethics code no. IR.MUI.REC.1396.3.938) .The study was carried out on 50 parents and their children with visual impairment referring to schools of the blind children (Shahid Abedi and Shahid Samani schools) in Isfahan in 2017- 2018. 

Visually impaired children aged 8-14 years, living in Isfahan and the children whose parents fulfilled informed consent, were enrolled in the study. Children with a history of any syndromic or mental disease, uncooperative children and Lack of response to more than 20 percent of the questions by parents excluded from the study.

After taking informed consent, awareness of parents was measured by parental knowledge questionnaire. The content validity of the questionnaire was assessed and approved by experts and professors in the related field (pedodontists, community oral health specialists, pediatricians). In order to confirm the reliability, the questionnaire was also given to twenty children in a pilot study. The Cronbach alpha was determined 0.81, indicating an acceptable reliability. 

The final questionnaire consisted of demographic information and 26 questions for assessing parental awareness in three areas of oral health, using oral health services and consequences of poor oral health.

The parents were asked to respond to the knowledge questions. The answers were scored based on the 5-point Likert scale ranging from 1 (completely disagree) to 5 (completely agree). The total score of each questionnaire shows how knowledgeable the parents were about oral health preventive care of their children.

The participant responses were ranked to “good,” “moderate” and “weak” categories. In a way that Individual scores ranging from 26-60, 61-95 and 96-130 were considered as weak, moderate, and good, respectively. After completing the questionnaire, educational pamphlets were provided to parents about the principles and methods of health care.

Then the DMFT index of first permanent molar was recorded by one examiner using mouth mirror, explorer and head light according to World Health Organization(WHO) [ 8 ] criteria. Data were analyzed using Spearman correlation coefficient, Pearson correlation and T-test in SPSS 22 software. The level of significance was considered less than 0.05.

## Results

A total of 50 visually impaired school children (54% boys, 46% girls) of Isfahan were enrolled in the study. The children age group was in the range of 8-14 years with the mean of 10.3±1.7. The study group consisted of 80% congenital and 20% acquisitive visual disorder. The mean age of parents participated in the study was 39.5±5.6 (min 30 years and max 50 years) wherein 76 % were mothers and 24% were fathers. Most of parents were housekeeper (52%) and diploma educated (34%). Mean score of parental awareness about oral health preventive care was 68.4±15.5 where in 31 (62%) and 19 (38%) of them had good and moderate awareness respectively. There was no significant relation between awareness score and parental age (*p*= 0.43). While awareness score had a significant direct relation with parental education (*p*= 0.04). There was no significant difference between parents in awareness score (*p*= 0.61). Also mean score of parental awareness between housewives and other occupations was not significantly different (*p*= 0.39).

Mean DMFT in the studied children was 2.40±1.32, wherein the highest score was related to decayed teeth with the mean of 1.74±1.26, followed by filled teeth. There was no significant relation between parental knowledge and mean DMFT (*p*= 0.30), while there was revers relation with number of extracted tooth (*p*= 0.02) and direct relation with number of filled tooth (*p*= 0.04). 

There was no significant difference between the two sexes in terms of decayed (*p*= 0.99), missed (*p*= 0.11), filled teeth (*p*= 0.71) and DMFT index (*p*= 0.64). There was no significant relation between children age and decayed (*p*= 0.47), missed (*p*= 0.33), filled teeth (*p*= 0.51) and DMFT index (*p*= 0.51) separately (Table 1).

**Table 1:** Pearson correlation of DMFT, D, M, and F with children age and Parental knowledge

Variable	Parental Knowledge		Age	
	Significance	Correlation (r)	Significance	Correlation (r)
D	0.57	0.081	0.47	-0.105
M	0.02	-0.283	0.33	0.141
F	0.04	0.249	0.51	0.097
DMF	0.30	0.151	0.72	0.053

## Discussion

Improving quality of life is considered as an indicator of rehabilitation in people especially with physical disabilities. The quality of life is greatly altered by oral health. Physically disabled children usually take a lot of care about their disabilities, but they do not care about their oral health [ 9 ]. Visually impaired children are more prone to have dental carries, periodontal diseases and more problems in accessing dental care. Oral health of the children is associated with oral health knowledge of their parents [ 10 ]. By increasing parental awareness, the incidence of dental caries and complicated dental treatments reduced [ 11 ].

According to study findings, the mean score of parental awareness score was 68.4±15.5 wherein 31 (62%) and 19 (38%) of them have good and moderate awareness score respectively. As a result the awareness of the parents of visually impaired children about the preventive care was moderately high. This finding is in accordance with the study of Shirani *et al*. [ 12 ] which evaluated the knowledge, attitude, and practice of mothers visiting dental clinics in Isfahan wherein 44% and 44.7% had good and moderate awareness score respectively. In recent years, it seems that more informing schools and kindergartens are raising awareness of parents in this regard.

Mean DMFT in the study population was 2.40± 1.32, wherein the highest score was related to decayed tooth. Hence the mean DMFT of first permanent molar and especially decayed teeth in visually impaired children was moderately high. Tahani *et al*. [ 13 ] also revealed that dental caries status in visually impaired children was above the state and province norms in the 4‒12-years old children. Parkar *et al*. [ 14 ] demonstrated that visually impaired individuals have moderate to low grade of oral hygiene status, with very high rate of caries prevalence. Shetty *et al*. [ 15 ] illustrated there was an overall increase in the prevalence of dental caries among visually impaired children. Eskandarian *et al*. [ 16 ] evaluated DMFT, OHI-S status and occlusion in 10-12 years old handicapped children of Shiraz and demonstrated that the worst oral hygiene status was related to visually impaired children. This can be due to inefficient tooth brushing and more plaque and calculus accumulation in visually impaired children. Reddy *et al*. [ 17 ] illustrated there was a greater prevalence of dental caries, poorer oral hygiene, and higher incidence of trauma in visually impaired children. In Ahmad *et al*. [ 18 ] study, most of the blind children had poor oral hygiene.

Visually disabled children, due to their inability to learn oral hygiene instructions, need more care of dentists and authorities in implementation of preventive and therapeutic programs. Oral hygiene instruction should be encouraged including tongue cleaning, twice daily brushing, and dental flossing that should be preferably done under supervision of care providers. 

Parental awareness generally did not show significant relationship with DMFT, while by increasing parental knowledge, the number of missed teeth was decreased and the number of filled teeth was increased in visually impaired children. Based on the study findings, as parental awareness is rising, they pay more attention to the importance of maintaining teeth.

Hence, by early detecting decayed teeth in their children mouth, the parents visit the dentist to repair the teeth and prevent further caries progression and early loss of their children teeth. Accordingly, formulation and implementation of a complete plan to raise awareness of parents and institutional dental treatment programs, especially in visually impaired children that are more vulnerable in this field, would be an effective step towards improving oral health. DMFT index had no significant relation with children age and gender in current study. Yousofi *et al*. [ 19 ] and Marasouli *et al*. [ 20 ] demonstrated that the caries index increased by age. Alimorad *et al*. [ 21 ] revealed that dental caries was more prevalent in girls. This discrepancy in results may be related to different study location and population, since cultural, racial, and economic conditions might influence the prevalence of caries. 

The mean of awareness score had no significant relation with parental age, gender and occupation, while, by increasing parental education their awareness increased. Moallemis *et al*. [ 22 ] and Shirani *et al*. [ 12 ] also report that maternal education was significantly related to their awareness.

In explaining this finding, it can be said that education is one of the most important socioeconomic indicators that affects the knowledge, attitude and skills of parents. People with higher education seem to have more access to different sources to obtain the information in oral health preventive care. The questions used in this study included basic information which could evaluate parental awareness of oral health in visually impaired children. However further studies with larger sample size and use of more accurate and detailed questions in this field may be useful to get more reliable evidence.

## Conclusion

Parental awareness generally did not show significant relationship with DMFT, while by increasing parental knowledge the number of missed teeth decreased and the number of filled teeth increased in visually impaired children. 
